# Hydrochar Loaded with Nitrogen-Containing Functional Groups for Versatile Removal of Cationic and Anionic Dyes and Aqueous Heavy Metals

**DOI:** 10.3390/w16233387

**Published:** 2024-11-25

**Authors:** Yue Zhang, Yongshan Wan, Yulin Zheng, Yicheng Yang, Jinsheng Huang, Hao Chen, Jianjun Chen, Ahmed Mosa, Bin Gao

**Affiliations:** 1 Department of Agricultural and Biological Engineering, University of Florida, Gainesville, FL 32611, USA; 2 US EPA Center for Environmental Measurement and Modeling, Gulf Breeze, FL 32561, USA; 3 Department of Agriculture, Landscape, and Environment, University of Vermont, Burlington, VT 05405, USA; 4 Mid-Florida Research & Education Center, Environmental Horticulture Department, University of Florida, Apopka, FL 32703, USA; 5 Soils Department, Faculty of Agriculture, Mansoura University, Mansoura 35516, Egypt; 6 Department of Civil and Environmental Engineering, Rensselaer Polytechnic Institute, Troy, NY 12180, USA

**Keywords:** hydrothermal carbonization, engineered hydrochar, heavy metals, nitrogen doping, surface functionalization

## Abstract

Developing novel sorbents for effective removal of heavy metals and organic dyes from industrial wastewater remains a central theme for water research. We modified hydrochar derived from the hydrothermal carbonization of wheat straw at 180 °C with 3-Aminopropyl triethoxysilane (APTES) to enhance its versatile adsorption of Pb(II), Cu(II), methylene blue (MB), and reactive red (Red). Pristine and modified hydrochar (HyC and APTES-HyC) were characterized and tested for sorption performance. Characterization results revealed an enriched presence of N-functional groups, mainly -NH_2_ and C-N, on APTES-HyC, in addition to an increased specific surface area from 1.14 m^2^/g (HyC) to 4.51 m^2^/g. APTES-HyC exhibited a faster adsorption rate than HyC, reaching equilibrium approximately 4 h after initiation. The Langmuir adsorption capacities of APTES-HyC were 49.6, 14.8, 31.7, and 18.3 mg/g for Pb(II), Cu(II), MB, and Red, respectively, about 8.5, 5.0, 1.3, and 9.5 times higher than for HyC. The enhanced adsorption performance of APTES-HyC is attributed to the increased N-functional groups, which facilitated adsorption mechanisms specific to the pollutant of concern such as formation of frustrated Lewis pairs and cation–π interactions for metal ions and π–π interactions and hydrogen bond for dyes. This study offers a novel and facile approach to the synthesis of N-doped carbon materials for practical applications.

## 1. Introduction

The escalation of industry and urbanization has led to a noticeable rise in environmental degradation. Heavy metals and organic dyes, which are often co-pollutants in industrial wastewaters generated in the pre-treatment, dyeing, and printing processes of the textiles, pharmaceutical, paper, and pulp industries [1], are among the most salient contaminants threatening the water environment [2,3]. Both groups of contaminants constitute a peril to the environment and human health, exerting an undeniable impact on sustainable development [4]. To grasp the magnitude of human industrial activities on the hydrosphere, about 400 × 106 tons of aqueous pollutants are discharged annually to water bodies [5].

Discharges from the textile printing and dyeing industries account for about 20% of the total generated wastewater worldwide, with a global production of about 80 × 106 tons containing approximately 10,000 types of non-biodegradable dyes, a blend of carbon-based organic compounds and pigments [6,7]. Methylene blue (MB) and reactive red dye (Red) represent the most common cationic and anionic dyes organic dyes, respectively. Elevated levels of heavy metals such as Pb(II) and Cu(II) are also frequently detected [8]. Pb(II) exposure poses serious risks to human health, particularly damaging the brain and nervous system in children [9]. In adults, Pb(II) exposure is associated with elevated blood pressure, impaired kidney function, and reproductive issues [10]. Additionally, excessive Cu(II) intake can lead to liver and kidney damage as well as symptoms such as nausea, vomiting, and diarrhea [11]. Effectively addressing the pollution from heavy metals and organic dyes has thus become a crucial societal imperative.

Wastewater treatment traditionally tackles heavy metals and organic dyes separately in the treatment process due to the fundamental differences between the two groups of contaminants [12,13]. Examples of treatment techniques include adsorption, chemical precipitation, electrostatic interaction, ion exchange, and membrane separation for heavy metals [14–17] and coagulation, electrolysis, and anaerobic hydrolysis to address organic dye contamination [18–20]. Adsorption is widely recognized as one of the most convenient, cost-effective, and eco-friendly techniques among these options [21–23]. Thus, it is crucial to design a sorbent that can effectively remove both heavy metals and organic dye contaminants to enhance the efficiency of wastewater treatment systems.

Biochar, a carbonaceous substance formed by the pyrolysis of biomass in an environment with limited oxygen, possesses a large specific surface area, great adsorption properties, and cost-effectiveness, rendering its use increasingly prominent in the domain of environmental remediation [24,25]. The synthesis of biochar can be adjusted to targeted applications based on the optimization of pyrolysis temperature, feedstock type, and engineering modification involving biological, physical, and chemical processes. Hydrothermal carbonization (HTC) is a milder treatment that utilizes water and lower-temperature conditions to convert biomass (e.g., plant residues, sludges) into a carbon-containing solid product known as hydrochar, a special type of biochar [26,27]. The HTC process is performed at low pressures (0.3–4.0 MPa), can be conducted within a temperature range of 150 °C to 375 °C, and the reaction time usually ranges from 4 to 24 h [28]. In the process of hydrothermal carbonization, biomass is combined with water or other liquids in a hydrothermal reactor, and subsequently, the hydrothermal reaction occurs to activate the hydrothermalized biomass, producing an inert biosorbent dominated by carbon [29]. Compared to conventional high-temperature carbonization, hydrothermal carbonization is carried out under milder conditions, and as a result, the product usually retains a considerable amount of organic compounds and oxygen-containing functional groups, contributing to functionalities of the product [30]. Although hydrochar has a relatively lower specific surface area and pore volume than biochar, its surface is highly aromatized, which contributes to its strong affinity for water [31]. Moreover, the production process for hydrochar is highly efficient and does not require pre-drying. The relatively low-temperature operation makes hydrochar an energy-efficient choice over biochar as an adsorbent [32]. Hydrothermal carbonization technology has potential applications in the fields of energy, ecology, and material sciences [33]. It can be applied to develop catalyst carriers, adsorbent materials, and environmental treatment materials thanks with unique properties such as uniform size, regular morphologies, and physical and chemical stability [34–36].

Hydrochar is a promising adsorbent for applications in wastewater treatment [37,38]. Typically, pristine hydrochar lacks sufficient adsorption sites for pollutants because it has a restricted specific surface area and pore volume [39]. It is widely recognized that functional groups on the surface of hydrochar controls contaminant adsorption [40]. Therefore, chemical and/or physical modification of hydrochar is often employed to boost the quantity and diversity of functional groups as a means to improve its contaminant sorption performance [41–43]. Prior research has demonstrated that it is possible to accomplish surface functionalization of hydrochar using oxidizing agents, including potassium permanganate (KMnO_4_) or hydrogen peroxide (H_2_O_2_), which introduce O-containing functional groups [29,44]. Comparable studies on loading N-containing functional groups on hydrochar for the sorption of both heavy metal and dye pollutants are limited in the literature.

In this study, we employ 3-Aminopropyl triethoxysilane (APTES) to functionalize a wheat straw hydrochar (HyC), thereby increasing the sorption of N-containing functional groups onto the hydrochar. Previous studies have shown that APTES-modified graphene oxide significantly enhances the adsorption capacity for organic pollutants and improves the adsorbent’s reusability [45,46]. Based on the functioning of N species in hydrochar, we hypothesize that APTES modification increases adsorption of both cationic and anionic dyes and aqueous heavy metals. To test our hypothesis, we follow the functionalization process to obtain a modified hydrochar (APTES-HyC) material, and further characterize and thoroughly test the adsorbents for their performance in adsorbing Pb(II), Cu(II), MB, and Red. We also elucidate the adsorption mechanisms in terms of interactions between N-containing functional groups with each contaminant tested. Ultimately, this study renders a novel and straightforward method for the synthesis of N-doped carbon materials for practical environmental applications.

## 2. Materials and Methods

### 2.1. Materials

Wheat straw was obtained locally and used as feedstock to produce hydrochar. All chemicals, including 3-Aminopropyl triethoxysilane (APTES, 99%), Pb(NO_3_)_2_, Cu(NO_3_)_2_·3H_2_O, MB (C_16_H_18_CIN_3_S), and Red (C_44_H_30_C_l2_N_14_O_20_S_6_), were of analytical grade, ACS-certified, and obtained from Thermo Fisher Scientific (Waltham, MA, USA). Deionized (DI) water used in this study had a conductivity of 0.055 μS/cm.

### 2.2. Sorbents Preparation

Wheat straw was first washed, air-dried, and then ground to a particle size of 0.5–1 mm. Subsequently, 20 g of the finely ground wheat straw was mixed with 200 mL of DI water and placed into a 500 mL hydrothermal reactor. The reactor was heated to 180 °C and maintained at this temperature for 4 h. The resultant blend was subjected to vacuum filtration to segregate the product. The solid was rinsed with DI water and dried at 80 °C for 24 h. The pristine wheat straw hydrochar was named HyC.

To produce modified hydrochar, 20 g of milled wheat straw was first mixed with 30 mL APTES (purity 97%) and 170 mL DI water and sonicated for 3 h. It was then transferred into a 500 mL hydrothermal reactor. The reactor was sealed and its temperature raised to 180 °C for 4 h. Next, the solid was separated by vacuum filtration, followed by rinsing with DI water, and finally dried at 80 °C for 24 h. This modified wheat straw hydrochar was named APTES-HyC. The yield of HyC obtained during this process was 79%, whereas the yield of APTES-HyC was 73%.

### 2.3. Characterization of Hydrochar

The hydrochar samples were characterized using the following metrics before and after APTES modification. The specific surface area was analyzed using the Brunauer–Emmett–Teller (BET) technique. The surface arrangement was examined by scanning electron microscopy (SEM, Hitachi SU8020, Tokyo, Japan) and energy-dispersive spectroscopy (EDS). The alterations in functional groups were detected by Fourier transform infrared spectroscopy (FTIR, Nicolet IS 10, Waltham, Massachusetts, USA) and X-ray photoelectron spectroscopy (XPS, Thermo ESCALAB 250Xi, Waltham, Massachusetts, USA).

### 2.4. Sorption Characteristics of Heavy Metals and Dyes

Batch tests were conducted at room temperature to examine the sorption kinetics and isotherm of Pb(II), Cu(II), MB, and Red using HyC and APTES-HyC. The sorption kinetics of Pb(II) and Cu(II) were investigated by introducing 100 mg sorbent into a 50 mL solution of 150 mg Pb(II)/L or 30 mg Cu(II)/L. The specimens were subsequently agitated on a shaker at 230 rpm for a duration ranging from 5 min to 48 h. The solution was filtered with a Fisherbrand^®^ 0.45 μm nylon membrane, and Pb (II) or Cu(II) levels were determined employing inductively coupled plasma emission spectrometry (ICP-OES, Spectro Blue, Kleve, Germany). To study the sorption isotherm, 100 mg of sorbent was introduced into 50 mL Pb(II) or Cu(II) solutions with initial concentrations ranging from 5 to 150 mg/L for Pb(II) or 5 to 50 mg/L for Cu(II). The specimens were agitated at 230 rpm for 24 h, filtered, and metal concentrations were measured as described earlier.

For dye sorption experiments, MB and Red stock solutions (200 mg/L) prepared using DI water were diluted to beginning concentrations ranging from 10 to 200 mg/L. The adsorption kinetics were conducted by introducing 100 mg of sorbent into 50 mL 100 mg/L MB or 50 mg/L Red solutions. The specimens were subsequently stirred on a shaker at 230 rpm for a duration ranging from 5 min to 48 h. After filtration with Fisherbrand^®^ 0.22 μm filter, MB and Red concentrations in the filtrates were determined using a UV spectrophotometer at 665 nm and 515 nm, respectively. The adsorption isotherm was generated by combining 100 mg sorbent with 50 mL MB or Red dye solutions with starting concentrations varying from 10 to 200 mg/L. After 24 h agitation on a shaker at 230 rpm, the mixtures were similarly filtered and measured for MB and Red in the filtrates. The experimental procedure was conducted under neutral pH and room temperature conditions.

The EPA quality assurance and quality control (QA/QC) standard procedure was performed for testing instruments (ICP-OES), preparing the calibration standards, measuring laboratory blanks, and sample replicants such as serial dilutions to ensure reliable, consistent, and accurate results [47]. All adsorption experiments were conducted in duplicate, and the average results were provided. A parallel control experiment was conducted under identical conditions without the presence of any sorbent material.

### 2.5. Mathematical Models

The pseudo-first-order, pseudo-second-order, and Elevich models were applied to simulate the sorption kinetics, and the Langmuir and Freundlich equations were used to model sorption isotherms. Detailed descriptions of these models can be found in the Supporting Information.

## 3. Results and Discussion

### 3.1. Characterization of Hydrochar

#### 3.1.1. SEM and EDS

**Figure 1.**
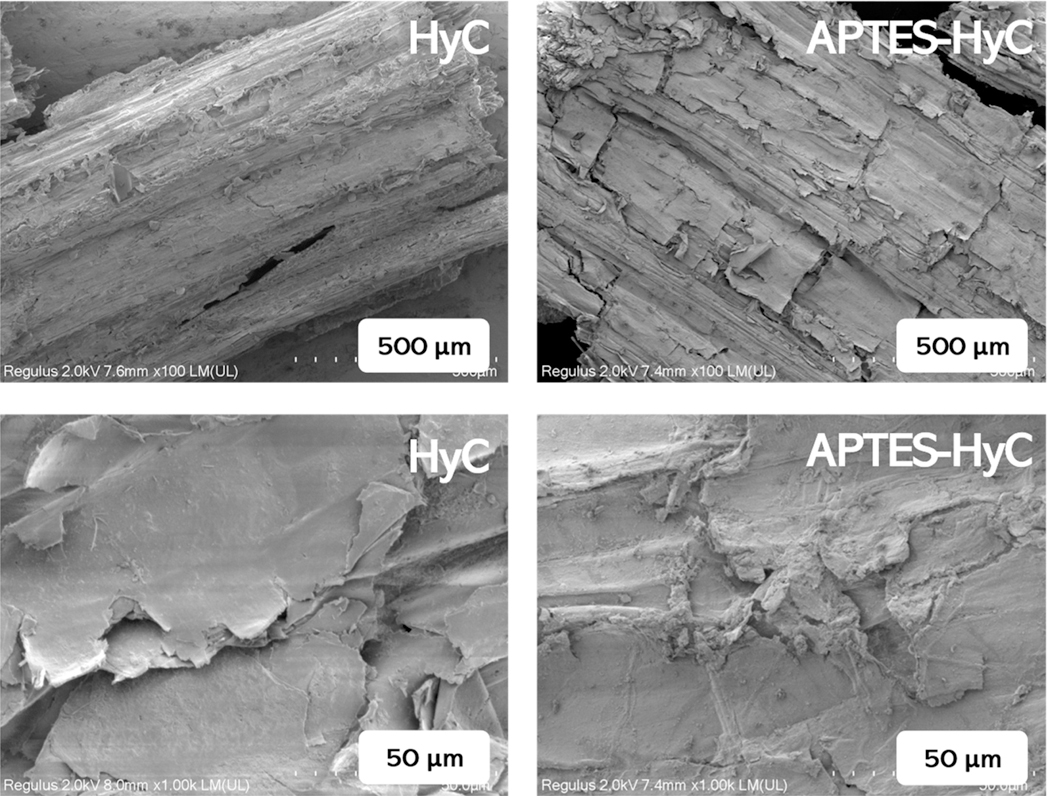
SEM images of HyC and APTES-HyC.

**Figure 2.**
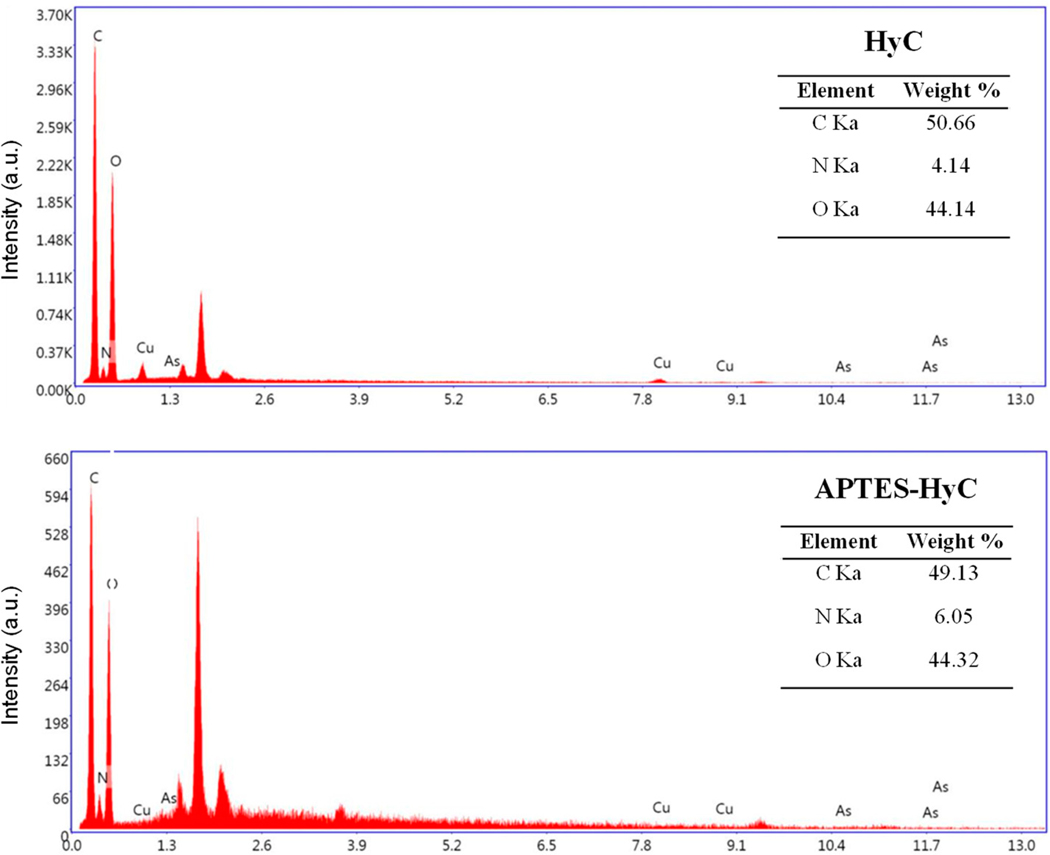
EDS spectra of HyC and APTES-HyC.

Figure 1 displays the morphology of HyC and APTES-HyC, exhibiting the partial retention of the original wheat straw structure in HyC. The application of APTES caused the surface texture and pores of HyC to be disturbed, and a homogeneous multilayer structure was observed, thus facilitating the generation of active sorption sites. However, the specific surface area increased only from 1.14 m^2^/g (HyC) to 4.51 m2/g (APTES-HyC) after modification. This change is considered minimal when compared with other activation or oxidation procedures [44,48]. The surfaces modified with APTES contain different numbers of dark spots, which are polymerized aminosilane molecules in the form of islands. The EDS spectra (Figure 2) exhibited N content in the hydrochar surface, which increased from 4.14% in HyC to 6.05% in APTES-HyC.

#### 3.1.2. FTIR

**Figure 3.**
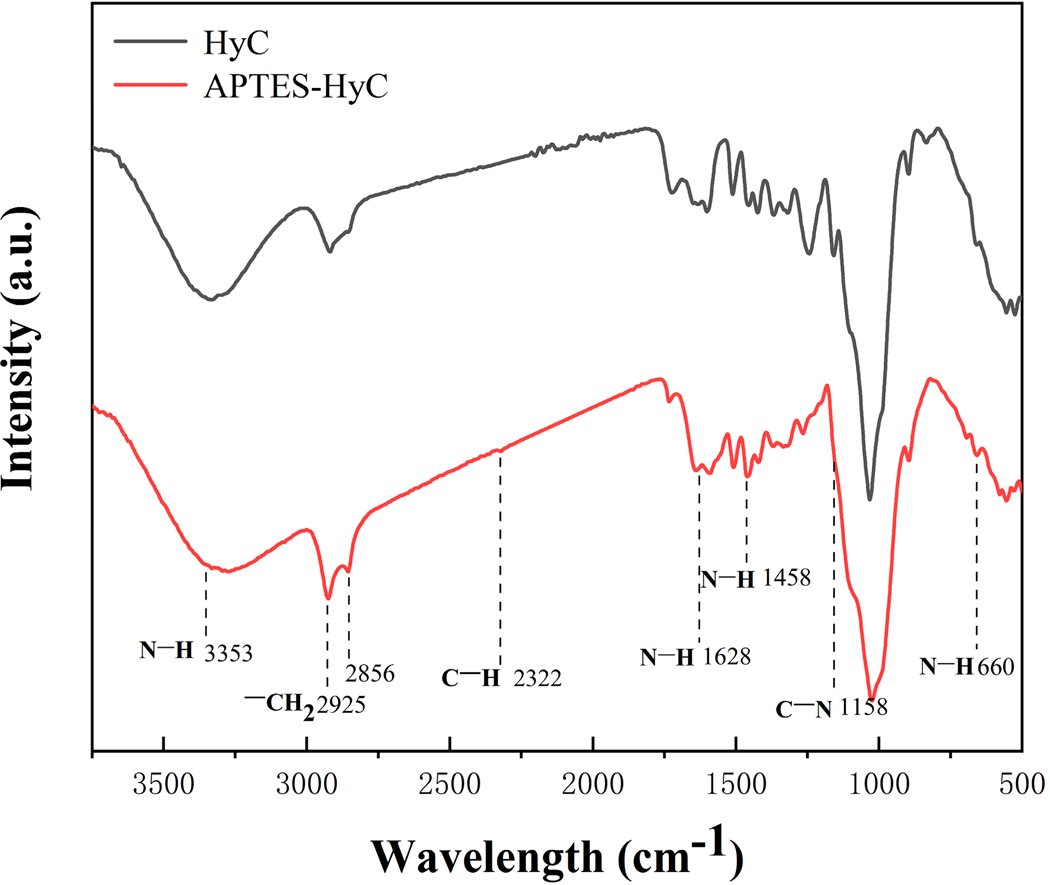
FTIR spectra of HyC and APTES-HyC.

The FTIR spectra (Figure 3) depict the changes in functional groups on hydrochar before and after modification. The wide spectral band seen at 3353 cm^−1^ can be attributed to the symmetric stretching of the N-H band and -OH stretching vibration [49]. Confirmation of the presence of APTES propyl groups was attained through the observation of the C-H stretching vibration occurring at 2322 cm^−1^ [50]. In addition, the spectral features detected at peaks 2925 and 2856 cm^−1^ substantiated the presence of propyl chains in APTES, denoted by the -CH_2_ stretching mode [51]. Compared to the pristine hydrochar, the magnitude of the N-H peak increased following N doping. With N doping, a small peak emerged at 1158 cm^−1^, which can be ascribed to the amine’s C-N stretching vibration. The NH_2_ group’s energy bands within the APTES molecule engage with the O-H stretching bands found in the hydrochar, resulting in the emergence of combined energy bands spanning from 1210 to 1228 cm^−1^ [52]. The spikes at 1628 cm^−1^ and 1458 cm^−1^ are associated with the NH_2_ deformation modes of the amine groups. These amine groups are highly hydrogen bound to the silanol groups, resulting in the creation of cyclic structures [53]. Furthermore, the N–H bending associated with the band at 660 cm^−1^ is observed in APTES-HyC. These findings confirm that the APTES modification has introduced N-containing functional groups to hydrochar.

#### 3.1.3. XPS

**Figure 4.**
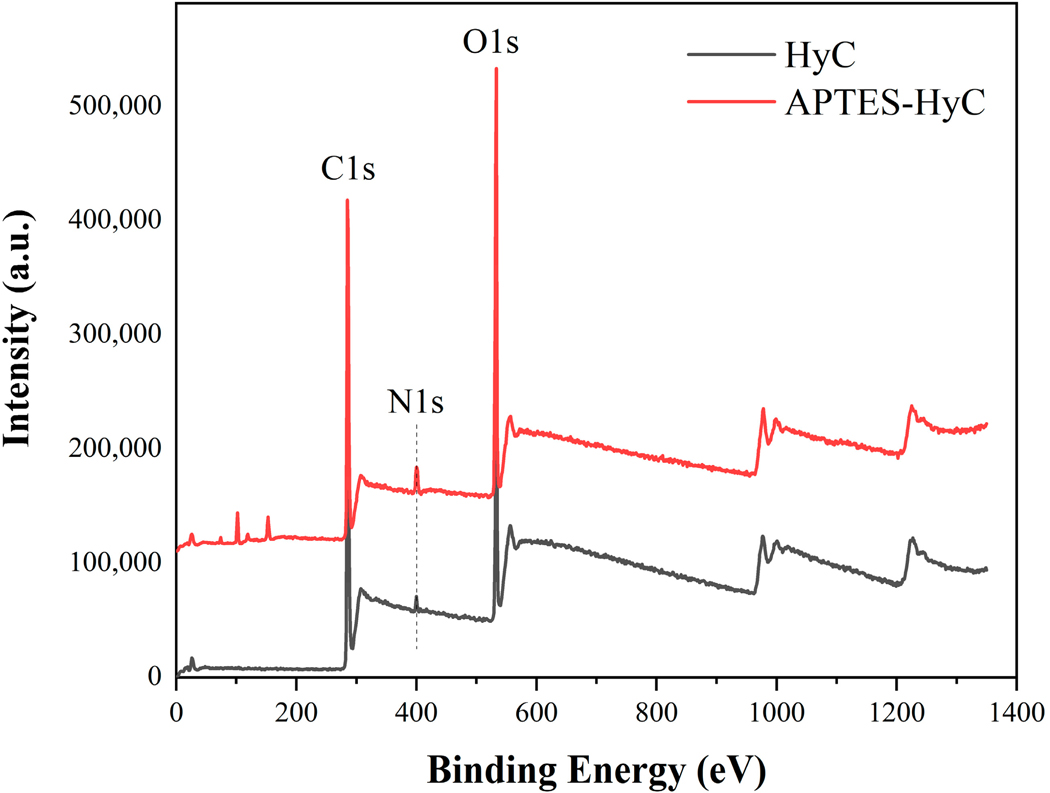
XPS spectra of HyC and APTES-HyC.

**Figure 5.**
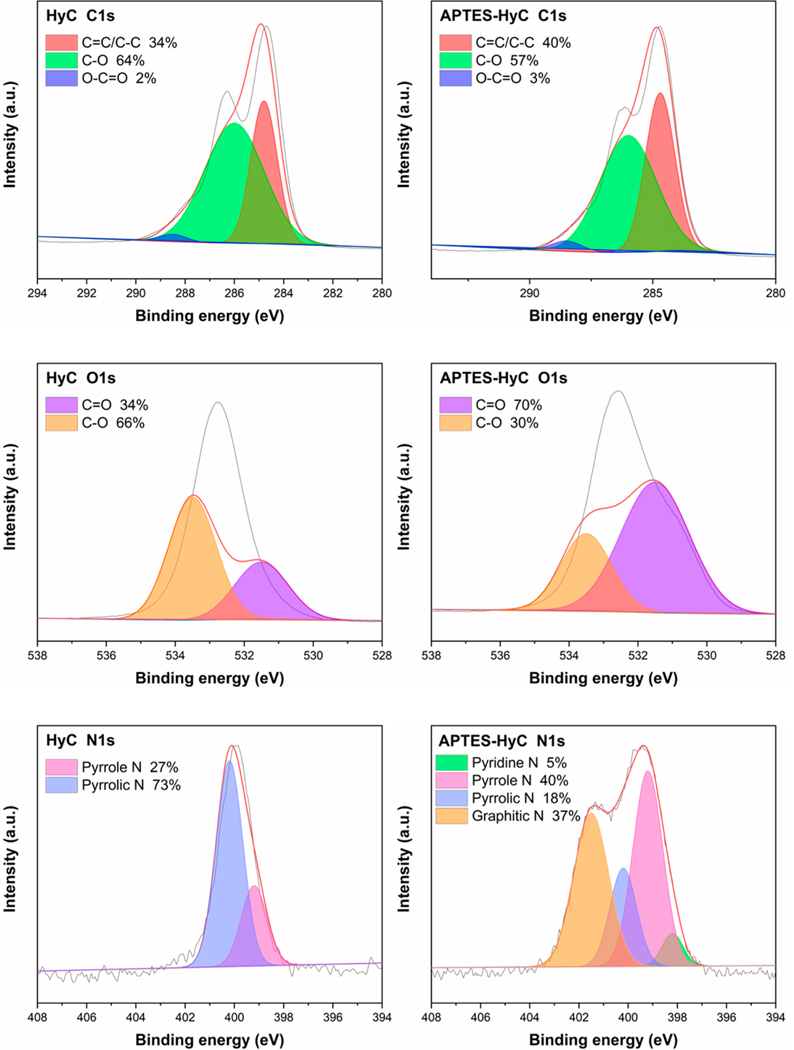
XPS spectra of C1s, O1s, N1s of HyC and APTES-HyC.

Figures 4 and 5 display the XPS spectra of the changed surfaces with high energy resolution. More precisely, there was a reduction in C1s from 73.11% in HyC to 68.63% in APTES-HyC. On the other hand, O1s rose from 25.56% in HyC to 27.77% in APTES-HyC, while N1s grew from 1.34% in HyC to 3.6% in APTES-HyC. This change is induced by the addition of the N-containing functional groups onto the modified hydrochar [54]. According to Figure 5, the C1s spectra exhibit three distinct peaks at about ~284.8, ~286.0, and ~288.5 eV, corresponding to the C=C/C–C, C-O, and O-C=O groups, respectively. The O1s spectra exhibit two peaks, C-O at ~533.0 eV and C=O at ~531.5 eV. The XPS spectra of hydrochar following N doping exhibited a prominent peak corresponding to the N1s orbital. The N1s spectra were deconvoluted into four peaks corresponding to graphitic N at ~401.5 eV, pyrrolic N at ~400.2 eV, pyrrole N at ~399.2 eV, and pyridine N at ~398.2 eV. The APTES-modified hydrochar exhibits the largest proportion of graphitic-N, which may contribute electrons to the conductive π-system and thereby increase its electrical conductivity [55,56]. The spectra of APTES-HyC showed a peak corresponding to N1s, suggesting the presence of significant N-containing functional groups on its surface.

### 3.2. Sorption of Heavy Metals and Dyes

#### 3.2.1. Sorption Kinetics

**Figure 6.**
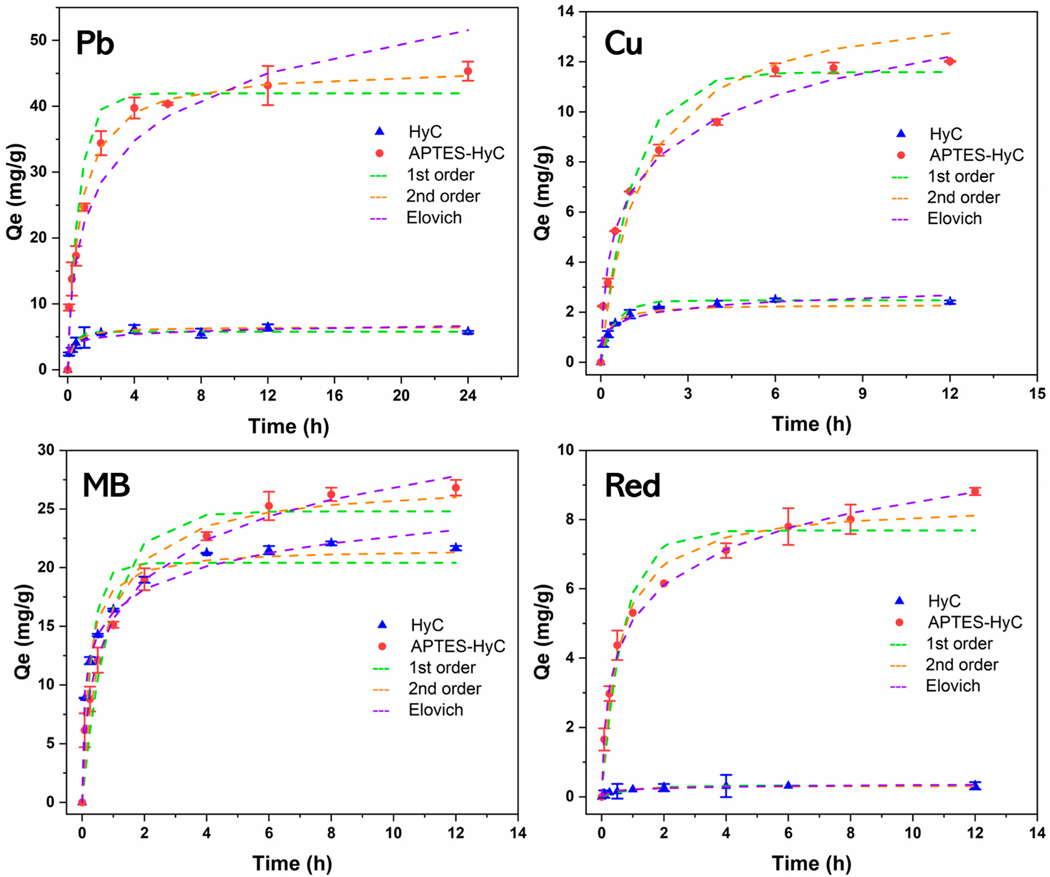
Sorption kinetics data and fitted models of Pb (II), Cu(II), MB, and Red onto HyC and APTES-HyC.

The sorption of Pb (II), Cu(II), MB, and Red on HyC and APTES-HyC exhibited comparable kinetics. Rapid adsorption occurred within the initial 2 h and thereafter slowed down. After 4 h, the adsorption reached a plateau (Figure 6), consistent with previous work [30,57]. N-doped hydrochar showed higher sorption capacity for Pb (II), Cu(II), MB, and Red than its pristine counterpart, likely in association with the introduction of -NH_2_ and -NH functional groups following APTES treatment. A mathematical description of the kinetic sorption behavior was performed using the pseudo-first-order, pseudo-second-order, and Elovich models. The resulting parameters associated with these models are reported in Table 1. The sorption kinetics were most accurately characterized by the pseudo-second-order model or Elovich model. The model simulations revealed a strong agreement with the experimental results with coefficient of determination (*R*^*2*^) values > 0.9 for all hydrochars. This finding suggests that the sorption kinetics of Pb (II), Cu(II), MB, and Red on the hydrochar samples are dominated by chemical adsorption involving multiple rate-determining steps or mechanisms.

#### 3.2.2. Sorption Isotherms

**Figure 7.**
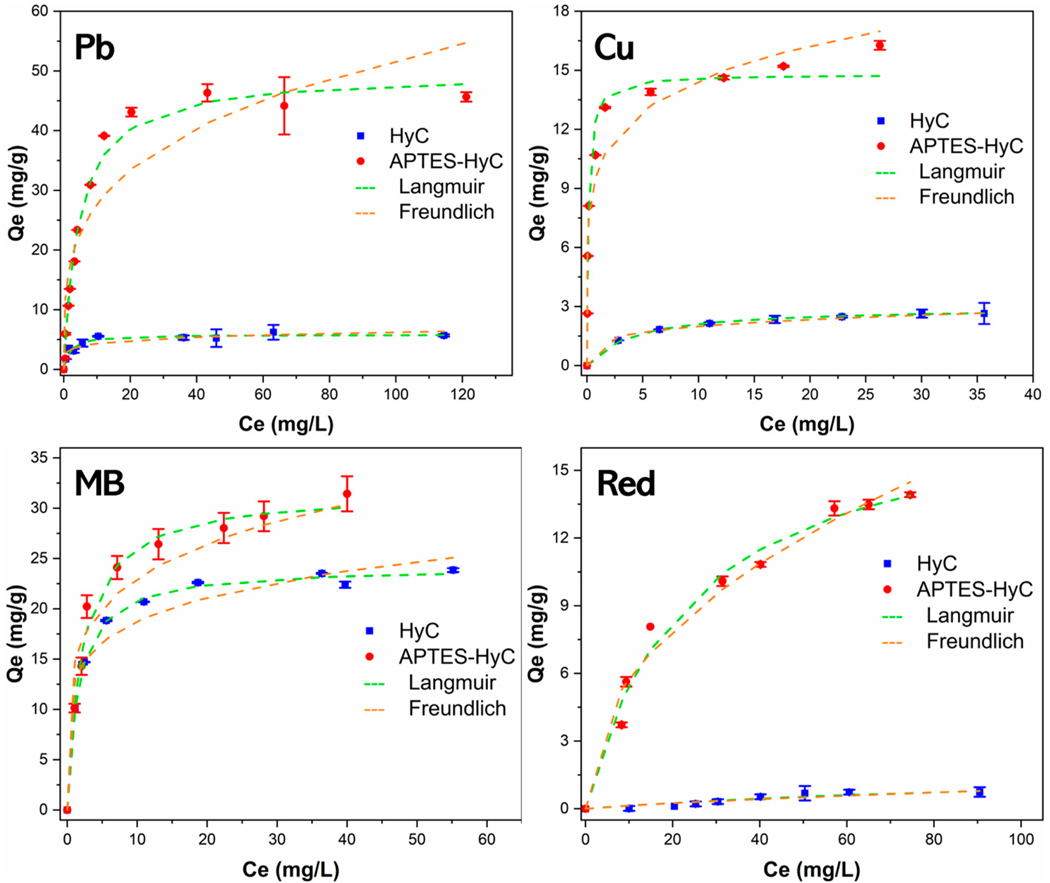
Sorption isotherm data and fitted models of Pb (II), Cu(II), MB, and Red onto HyC and APTES-HyC.

The experimental data of HyC and APTES-HyC were analyzed with the Langmuir model and Freundlich model, respectively (Figure 7). The Langmuir model provided a better fit than the Freundlich model (*R*^*2*^ values in Table 1), suggesting that APTES-HyC exhibits chemical homogeneity. The Langmuir maximum adsorption capacity of APTES-HyC was 49.62 mg/g for Pb(II), 14.79 mg/g for Cu(II), 31.70 mg/g for MB, and 18.31 mg/g for Red, about 8.5, 5.0, 1.3, and 9.5 times greater than for HyC, in association with the higher quantity of N-containing functional groups on the modified hydrochar.

Note that there are several related studies in the literature focusing on adsorbent functionalization capable of removing heavy metals and cationic organic dyes. For instance, alkali etching, which aims to increase oxygen-containing functional groups, was employed to functionalize hydrochar prepared from Chinese medicine industry waste, leading to adsorption capacities of 43 mg/g for Pb(II) and 31.1 mg/g for MB [58]. In another study with oxone-treated hydrochar to enhance carboxylic functionalities [59], the adsorption capacities reached 86.7 mg/g for MB and 46.7 mg/g for Pb(II). In contrast, the nanocomposite of hydrochar-Mg/Al LDH (layered double hydroxide) exhibits efficient removal for MB and Pb(II), achieving adsorption capacities of 256.54 mg/g and 33.55 mg/g, respectively [60]. Similarly, pomegranate-peel-derived hydrochar loaded with iron nanoparticles has been studied for the sorption of Cu(II) and MB, demonstrating adsorption capacities of 95.24 mg/g and 278 mg/g, respectively [61]. The adsorption capacities of a pristine rice husk hydrochar synthesized at a hydrothermal temperature of 220 °C are 9.7 mg/g for MB and 64.4 mg/g for Cu(II) [62]. Apparently, the adsorption capacity changes with feedstock materials, hydrothermal carbonization parameters, and functionalization methods. The method in this study aimed to increase nitrogen-containing functional groups, resulting in comparable adsorption capacities with the above studies. The versatile sorption of heavy metals, cationic organic dyes, and anionic organic dyes exhibited by APTES-HyC is a unique functionality seldom examined in the literature. Also note that functionalization methods resulting in high surface areas (such as hydrochar composites with LDH or ion nanoparticles) result in the higher adsorption capacity of MB. This disparity is indicative of an opportunity for improvement in the selection of modified materials or modification methods, demonstrating that considerable research is still needed for developing adsorbents capable of simultaneously removing heavy metals and organic dyes. The adsorption performance of APTES-HyC can be enhanced, based on previous studies, through acid-washing treatment or by extending the reaction time. For instance, the feedstock can be pretreated with 0.1 M HCl before APTES modification, or it can be added to an APTES solution with continuous heating and stirring for 6 to 12 h [45,50,63].

#### 3.2.3. Sorption Mechanisms

**Figure 8.**
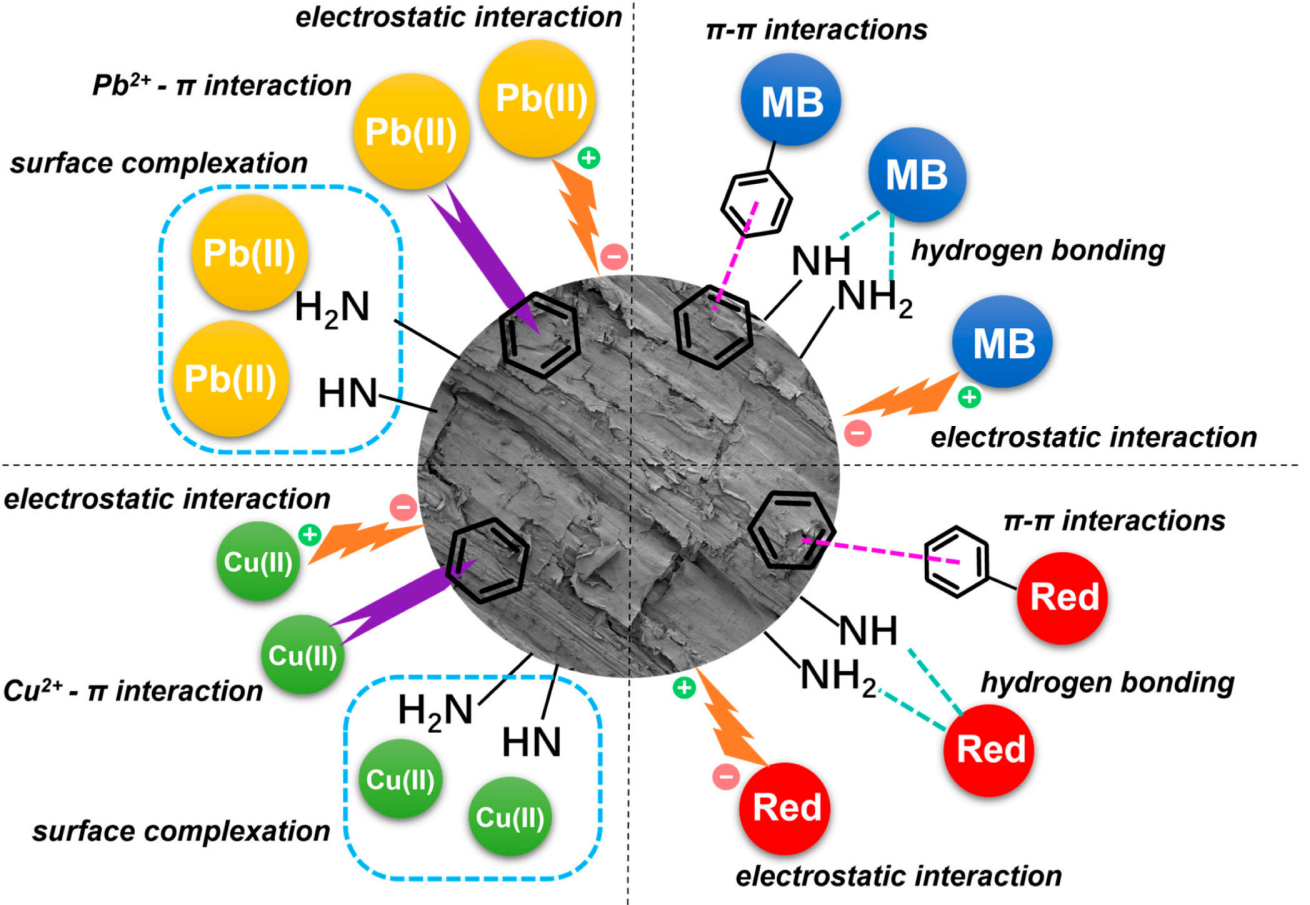
The proposed mechanisms for removal Pb (II), Cu(II), MB, and Red by APTES-HyC.

Figure 8 shows a schematic representation of the adsorption mechanisms for each contaminant. With the abundant N functional groups on APTES-HyC (mostly -NH and -NH_2_) as revealed by FTIR and XPS analyses, our focus is to discuss how they facilitate these adsorption mechanisms with respect to the unique characteristics of Pb(II), Cu(II), MB or Red. Various interactions may occur between heavy metal ions and the carbon surface, including the formation of surface complexes [64], ion exchange processes facilitated by strong surface acidic groups [65], and redox reactions associated with changes in the metal oxidation states [66]. For the modified hydrochar, the nitrogen atom of the amino group possesses free lone pair electrons (Figure 8). These electron-rich N species can interact with metal ions, demonstrating notable adsorption for heavy metals via robust chemical complexation and formation of frustrated Lewis pairs. The stabilized divalent electrostatic forces of Pb(II) and Cu(II) result in strong binding affinity of N atoms for Pb(II) and Cu(II) ions. The 37% graphitic-N present in APTES-HyC may also contribute to cation-π interactions [67,68], which are strengthened by the electron donating substituent, for example, amino -NH_2_ in the aromatic ring. The differential adsorption capacities of APTES-HyC for Pb(II) and Cu(II) under identical environmental conditions are likely attributable to variations in the ionic properties, including the ionic radius and hydration radius size [69]. The hydration radius of Pb(II) (4.01 Å) is comparatively smaller than that of Cu(II) (4.19 Å), whereas its ionic radius (1.20 Å) exceeds that of Cu(II) (0.72 Å) [70]. The adsorption capacity of metals increases as their hydration radius decreases. Metals with smaller hydration radii exhibit greater affinity for the adsorption process. The larger hydrated ions encounter difficulty in approaching the central adsorption site. Consequently, Cu(II), possessing a larger hydration radius, demonstrates a lower likelihood of adsorption to the active site of APTES-HyC than Pb(II), which has a smaller hydration radius [71,72]. Meanwhile, Pb(II), characterized by a larger ionic radius, tends to accumulate more readily on the surface and facilitate interaction between APTES-HyC and the aqueous solution [73].

The adsorption of MB as a cationic dye on carbon materials depends mainly on surface chemistry and electrostatic attraction. The presence of the N-O bond on APTES-HyC indicates that the adsorbed MB molecule has formed a chemical bond extending to the active site. Due to its high electronegativity of pristine hydrochar, the incorporation of N atoms results in a reduction of the electron density on the surface of the carbon material. Consequently, the π–electron acceptance capability of the modified hydrochar is enhanced (Figure 8). The N functional groups may work as electron donor while the aromatic ring in MB can function as an electron acceptor, facilitating the electron donor–acceptor mechanism. Thus, π–π interactions may play a key role in the adsorption of MB onto APTES-HyC. However, the enhancement over its pristine counterpart is not as significant as for Red, possibly because MB also contains protonated amide groups. Previous studies have established that an increased specific surface area enhances the adsorption of MB [44]. In this study, the surface area changed little before and after APTES modification, resulting in relatively less improvement of MB adsorption capacities. Finally, the -OH group of MB may form a hydrogen bond with N functional groups on APTES-HyC.

Red is a representative anionic dye, which contains negatively charged SO^−3^ acidic groups in its chemical structure, and pristine hydrochar typically exhibits a low adsorption capacity for such dyes. However, modification with APTES introduces N-containing functional groups, such as -NH and -NH_2_, onto the hydrochar, facilitating electrostatic adsorption of contaminants with negative charges. These functional groups interact electrostatically with the negative charges of Red, contributing to the significantly different adsorption capacity between HyC and APTES-HyC (Figure 8). Another adsorption mechanism, hydrogen bonding, is evident in the potential for dipole–dipole interactions between the hydrogen atoms on the surface of APTES-HyC and the O and N atoms within the Red dye structure [74]. Also, there is a mechanism wherein the benzene ring of the Red molecule interacts with the benzene ring in APTES-HyC through π–π interactions [75]. Consequently, Red is adsorbed onto the surface of APTES-HyC through a combination of electrostatic interactions, hydrogen bonding, and π–π interactions [76–78].

## 4. Conclusions

We can now conclude that a novel synthesis of N-doped hydrochar is successfully achieved by modifying pristine hydrochar with 3-Aminopropyl triethoxysilane. The N species introduced to the surface of the modified hydrochar are mainly amine (-NH_2_) and nitrile (C-N). These N groups play a key role in enhancing the sorption performance of hydrochar for Pb(II), Cu(II), methylene blue (cationic dye), and reactive red (anionic dye). The results of the batch adsorption experiments demonstrated that the adsorption of APTES-HyC proceeded rapidly within the first 2 h and reached equilibrium after 4 h. The maximum adsorption capacities were 49.62 mg/g for Pb(II), 14.79 mg/g for Cu(II), 31.70 mg/g for MB, and 18.31 mg/g for Red. This study provides a novel and facile approach to the synthesis of N-doped carbon sorbents for practical environmental applications.

## Supplementary Material

SI

## Figures and Tables

**Table 1.** Best-fit parameters of adsorption kinetic and isotherm models for Pb(II), Cu(II), MB, and Red adsorption onto HyC and APTES-HyC.

Adsorbate	Kinetic and Isotherm Models	Adsorbent	Parameter-1	Parameter-2	*R* ^2^

	Pseudo-first-order	HyC	*k*_1_ = 2.977	*q*_e_ = 5.776	0.845
		APTES-HyC	*k*_1_ = 1.422	*q*_e_ = 41.969	0.908
	Pseudo-second-order	HyC	*k*_2_ = 0.550	*q*_e_ = 6.509	0.876
		APTES-HyC	*k*_2_ = 0.030	*q*_e_ = 45.958	0.977
Pb(II)	Elovich	HyC	*α* = 480.670	*β* = 1.465	0.836
		APTES-HyC	*α* = 90.960	*β* = 0.106	0.913
	Langmuir	HyC	*K* = 0.645	*S*_max_ = 5.829	0.912
		APTES-HyC	*K* = 0.217	*S*_max_ = 49.622	0.985
	Freundlich	HyC	*K*_f_ = 3.024	*n* = 0.157	0.785
		APTES-HyC	*K*_f_ = 14.774	*n* = 0.273	0.834

	Pseudo-first-order	HyC	*k*_1_ = 2.041	*q*_e_ = 2.474	0.902
		APTES-HyC	*k*_1_ = 0.902	*q*_e_ = 11.593	0.926
	Pseudo-second-order	HyC	*k*_2_ = 2.000	*q*_e_ = 2.309	0.951
		APTES-HyC	*k*_2_ = 0.049	*q*_e_ = 14.679	0.927
Cu(II)	Elovich	HyC	*α* = 40.653	*β* = 2.677	0.940
		APTES-HyC	*α* = 42.560	*β* = 0.445	0.980
	Langmuir	HyC	*K* = 0.253	*S*_max_ = 2.959	0.993
		APTES-HyC	*K* = 7.203	*S*_max_ = 14.789	0.955
	Freundlich	HyC	*K*_f_ = 1.180	*n* = 0.228	0.950
		APTES-HyC	*K*_f_ = 9.963	*n* = 0.163	0.896

	Pseudo-first-order	HyC	*k*_1_ = 3.249	*q*_e_ = 20.397	0.784
		APTES-HyC	*k*_1_ = 1.114	*q*_e_ = 24.799	0.903
	Pseudo-second-order	HyC	*k*_2_ = 0.237	*q*_e_ = 21.639	0.931
		APTES-HyC	*k*_2_ = 0.056	*q*_e_ = 27.426	0.963
MB	Elovich	HyC	*α* = 898.887	*β* = 0.356	0.975
		APTES-HyC	*α* = 110.397	*β* = 0.201	0.993
	Langmuir	HyC	*K* = 0.630	*S*_max_ = 24.147	0.992
		APTES-HyC	*K* = 0.467	*S*_max_ = 31.691	0.978
	Freundlich	HyC	*K*_f_ = 12.715	*n* = 0.169	0.876
		APTES-HyC	*K*_f_ = 14.517	*n* = 0.200	0.869

	Pseudo-first-order	HyC	*k*_1_ = 1.000	*q*_e_ = 0.323	0.888
		APTES-HyC	*k*_1_ = 1.455	*q*_e_ = 7.686	0.906
	Pseudo-second-order	HyC	*k*_2_ = 8.000	*q*_e_ = 0.316	0.965
		APTES-HyC	*k*_2_ = 0.227	*q*_e_ = 8.469	0.971
Red	Elovich	HyC	*α* = 2.145	*β* = 17.472	0.964
		APTES-HyC	*α* = 43.435	*β* = 0.665	0.995
	Langmuir	HyC	*K* = 0.008	*S*_max_ = 1.931	0.849
		APTES-HyC	*K* = 0.042	*S*_max_ = 18.305	0.970
	Freundlich	HyC	*K*_f_ = 0.025	*n* = 0.770	0.814
		APTES-HyC	*K*_f_ = 1.989	*n* = 0.461	0.956

## Data Availability

Data available on request due to restrictions eg privacy or ethical. The data presented in this study are available on request from the corresponding author.
