# Supplementary material for: Hydrochar Loaded with Nitrogen-Containing Functional Groups for Versatile Removal of Cationic and Anionic Dyes and Aqueous Heavy Metals
Source: Water (Basel). Author manuscript; Available in PMC 2025 Dec 1. (PMC12181946; doi:10.3390/w16233387)
Supplement: SI [file NIHMS2069998-supplement-SI.pdf]

Supporting information

Hydrochar Loaded with Nitrogen-Containing Functional Groups for Versatile Removal of Cationic and Anionic Dyes and Aqueous Heavy Metals

Yue Zhang [1], Yongshan Wan [2,*], Yulin Zheng [1], Yicheng Yang [1], Jinsheng Huang [1], Hao Chen [3], Jianjun Chen [4], Ahmed Mosa [5] and Bin Gao [6]

[1] Department of Agricultural and Biological Engineering, University of Florida, Gainesville, FL 32611, USA
[2] US EPA Center for Environmental Measurement and Modeling, Gulf Breeze, FL 32561, USA
[3] Department of Agriculture, Landscape, and Environment, University of Vermont, Burlington, VT 05405, USA
[4] Mid-Florida Research & Education Center, Environmental Horticulture Department, University of Florida, Apopka, FL 32703, USA
[5] Soils Department, Faculty of Agriculture, Mansoura University, Mansoura 35516, Egypt
[6] Department of Civil and Environmental Engineering, Rensselaer Polytechnic Institute, Troy, NY 12180, USA
* Correspondence: wan.yongshan@epa.gov

Mathematical Models

The pseudo-first-order (eq. S1), pseudo-second-order (eq. S2), and Elevich model (eq. S3) were applied to simulate the sorption kinetics [79]:

$$q_t = q_e(1 - e^{-k_1 t}) \qquad \text{(S1)}$$

$$q_t = \frac{k_2 q_e^2 t}{1+k_2 q_e^2 t} \qquad \text{(S2)}$$

$$q_t = \frac{1}{\beta}\ln(\alpha\beta t + 1) \qquad \text{(S3)}$$

where q_t and q_e are the amount of sorbate removed at time t and at equilibrium, respectively (mg g^{-1}), and k_1 and k_2 are the first-order and second-order sorption rate constants (h^{-1}), respectively, α is the initial sorption rate (mg g^{-1}) and β is the desorption constant (g mg^{-1}). The first- and second-order models are semi-empirical equations, while the Elevich model is an empirical fitting equation.

Langmuir (eq. S4) and Freundlich (eq. S5) equations were used to fit measured sorption data. Langmuir model assumes that sorption occurs on homogeneous monolayer sorption without interactions among the solutes [80]. Uniform energy of sorption was assumed onto the surface and no transmigration of sorbate in the surface plane. Freundlich isotherm model assumes that sorption occurs on a heterogeneous surface [81]. The following equation represents them:

$$q_e = \frac{K S_{max} C_e}{1+K C_e} \qquad \text{(S4)}$$

$$q_e = K_f C_e^n \qquad \text{(S5)}$$

where K and K_f are the Langmuir bonding term related to interaction energies (L mg^{-1}) and the Freundlich affinity coefficient (mg$^{(1-n)}$ L^n g^{-1}), respectively, S_{max} is the Langmuir maximum capacity (mg kg^{-1}), C_e is the equilibrium solution concentration (mg L^{-1}) of the sorbate, and n is the Freundlich linearity constant.

References

79. Gerente, C.; Lee, V.; Cloirec, P.L.; McKay, G. Application of chitosan for the removal of metals from wastewaters by adsorption—mechanisms and models review. *Critical reviews in environmental science and technology* 2007, *37*, 41-127.

80. Langmuir, I. The adsorption of gases on plane surfaces of glass, mica and platinum. *Journal of the American Chemical society* 1918, *40*, 1361-1403.

81. Freundlich, H. Über die adsorption in lösungen. *Zeitschrift für physikalische Chemie* 1907, *57*, 385-470.
